# High Prevalence of Antimicrobial Resistance Genes in Rabbit Farms from Sumy Region, Ukraine

**DOI:** 10.3390/antibiotics14090907

**Published:** 2025-09-08

**Authors:** Sara Gomes-Gonçalves, Jaqueline T. Bento, Ana Machado, Yevheniia Dudnyk, Oksana Shkromada, Halyna Rebenko, Adriano A. Bordalo, João R. Mesquita

**Affiliations:** 1 School of Medicine and Biomedical Sciences (ICBAS), Universidade do Porto (UP), 4050-313 Porto, Portugal; 2 CIIMAR-Interdisciplinary Centre of Marine and Environmental Research, Universidade do Porto (UP), 4450-208 Matosinhos, Portugal; 3 Faculty of Veterinary Medicine, Sumy National Agrarian University, 40000 Sumy, Ukraine; 4 Associate Laboratory for Animal and Veterinary Science (AL4AnimalS), 1300-477 Lisboa, Portugal; 5 Centro de Estudos de Ciência Animal (CECA), Instituto de Ciências, Tecnologias e Agroambiente (ICETA), Universidade do Porto (UP), 4051-401 Porto, Portugal

**Keywords:** antimicrobial resistance genes, cuniculture, rabbits, Ukraine

## Abstract

**Background/Objectives**: Antimicrobial resistance (AMR) poses a significant threat to public health, food security, and sustainable livestock production. Despite increasing concern, AMR remains poorly studied in cuniculture, particularly in regions where rabbit farming is predominantly small-scale and household-based. This study aimed to assess the prevalence and diversity of antimicrobial resistance genes (ARGs) in rabbit farms in northeastern Ukraine. **Methods**: A total of 100 fecal samples were collected from clinically healthy rabbits housed in two farms and one vivarium. DNA was extracted and analyzed using real-time PCR targeting 21 ARGs associated with resistance to major antibiotic classes, including tetracyclines, sulfonamides, β-lactams, macrolides, quinolones, carbapenems, and multidrug efflux systems. **Results**: A diverse and widespread resistome was identified. The most prevalent genes included sul1 (96%), *blaTEM* (95%), *tetM* (94%), and *ermB* (93%). *AcrB* (78%) and *qnrS*/*oqxB* (approximately 67%) were also frequently detected. Carbapenemase genes (*blaKPC*, *blaNDM*, *blaVIM*) were identified in 6% of samples, despite limited veterinary use of carbapenems. Notably, 96% of samples harbored ARGs from three or more antibiotic classes, indicating a high potential for multidrug resistance. **Conclusions**: The findings demonstrate a high prevalence and diversity of ARGs in rabbit farming systems in northeastern Ukraine. The presence of clinically significant resistance genes, including those conferring carbapenem resistance, underscores the urgent need for targeted AMR surveillance and improved antibiotic stewardship in cuniculture, particularly in regions with minimal regulatory oversight of antimicrobial use.

## 1. Introduction

Antimicrobial resistance (AMR) is one of the most critical threats to global health, food safety, and sustainable animal production. It arises when microorganisms evolve mechanisms that render antimicrobials ineffective. This phenomenon severely compromises the ability to treat infections, leading to prolonged illness, increased mortality, and higher healthcare costs [1]. In addition to its impact on human health, AMR has major implications in the veterinary and agricultural sectors, particularly through the transmission of resistant pathogens from animals to humans via direct contact, food products, and environmental routes [2]. Recognizing its complex and interconnected nature, the issue of AMR has been placed at the center of the One Health approach, which highlights the need for integrated efforts across human, animal, and environmental health disciplines [3].

The use of antibiotics in veterinary medicine plays a crucial role in ensuring animal welfare and productivity. Antimicrobials are employed not only for therapeutic purposes but also as metaphylactic and prophylactic agents to prevent disease spread and onset, particularly in high-density and intensive farming systems [4]. On livestock farms, antibiotics have also been employed for non-therapeutic purposes such as enhancing growth and feed efficiency, leading to prolonged exposure of animals to low, sub-lethal concentrations, particularly in the gastrointestinal tract [5,6,7]. These conditions suppress susceptible bacteria while favoring the survival of resistant strains, accelerating the development and spread of AMR. The risk is further amplified by the chemical similarity between many veterinary antibiotics and those used in human medicine, increasing the potential for cross-resistance and posing significant public health concern [8].

Rabbit farming in Ukraine is mainly carried out by private households and backyards, which account for about 97% of production, mostly for family consumption, while only a small fraction (3%) is produced by agricultural enterprises [9,10]. In 2018, the country produced around 12 thousand tonnes of rabbit meat (slaughter mass), accounting for 0.5% of the total domestic meat production and placing Ukraine 8th in global rabbit meat production, contributing 1.1% of the total [6,9]. The development of organic rabbit meat farming presents a significant economic opportunity for the Ukrainian industry compared to other animal sectors [7,11]. Nevertheless, the rabbit farming sector faces critical challenges related to antimicrobial usage. Rabbits raised for meat in industrial systems exhibit the highest levels of antimicrobial consumption among food-producing animals, contributing to the emergence of AMR within the industry [12,13,14]. This is particularly concerning given rabbits’ high susceptibility to bacteria, which necessitates frequent antibiotic use, especially during stressful phases. The limited availability of veterinary medicines authorized specifically for rabbits often results in off-label and empirically guided antimicrobial use, further aggravating the risk of selecting multidrug-resistant bacteria [15]. These practices also raise environmental concerns, as resistance genes may be disseminated through fecal excretion and manure application. Despite the scale and intensity of rabbit farming, this sector remains underrepresented in AMR research and surveillance, and there is a notable lack of systematic data on antimicrobial use and resistance profiles, making it difficult to develop targeted mitigation strategies [9,16,17,18].

Understanding the use and impact of antibiotics in rabbit production is essential for developing evidence-based guidelines and interventions. This study aims to address this gap by investigating the administration, use practices, and resistance patterns associated with antimicrobial agents in rabbit farming. This work contributes to a broader understanding of AMR within animal production and highlights the need for species-specific surveillance and policy approaches.

## 2. Material and Methods

### 2.1. Sampling

In April 2024, 100 fresh fecal samples were collected from clinically healthy Californian domestic rabbits (*Oryctolagus cuniculus*), born and raised on two private farms (Farm 1 *n* = 9; Farm 2 *n* = 80) and in a vivarium (*n* = 11) inside a laboratory animal facility with uninfected animals in the Sumy region of northeastern Ukraine. Samples were collected directly from rabbit cages during routine cleaning, without handling or disturbing the animals, and transferred into individual sterile tubes to minimize the risk of contamination. They were immediately stored at 4 °C and transported to the laboratory within 12 h of collection. Upon arrival, samples were preserved at −20 °C until DNA extraction.

The vivarium housed clinically healthy rabbits kept in cages in separate rooms and fed with specialized commercial feed and purchased vegetables; these animals were not intended for meat production. Farm 1 was a small family-type with rabbits kept in individual cages, fed with commercial feed, farm-grown vegetables, and hay from local fields, with slaughtering carried out on-site for household use only. Farm 2 was a larger family-type holding, with rabbits kept in a separate building in three-tiered cages, fed with alfalfa, hay, wheat, and barley mixture purchased from local farmers, and farm-grown vegetables; slaughtering was also limited to household consumption.

Although the rabbits themselves were confined to cages, environmental exposure may occur indirectly through feed sourced from local fields that could be fertilized with animal manure. This sample set provides an illustrative example of how the degree of animal contact with the external environment may influence resistance.

### 2.2. DNA Extraction

Fecal samples (0.5 g) were suspended in 560 µL of Buffer AVL (QIAGEN, Venlo, The Netherlands) containing carrier RNA, thoroughly mixed, and incubated at room temperature for 10 min to ensure complete lysis and pathogen inactivation. The suspensions were subsequently centrifuged at 8000× *g* for 5 min, and 200 µL of the clarified supernatant was used for nucleic acid extraction. Total nucleic acids were isolated with the QIAamp Cador Pathogen Mini Kit (QIAGEN) on the QIAcube automated platform, following the manufacturer’s instructions. The purified DNA was eluted in RNase-free water and stored at −80 °C until further analysis.

### 2.3. Molecular Detection of AMR Genes

The molecular detection of antimicrobial resistance (AMR) genes targeted several antibiotic classes: Tetracycline resistance genes (*tetA*, *tetC*, *tetM*, and *tetW*), Carbapenemases resistance genes (*blaNDM*, *blaVIM*, *blaKPC*), β-lactamase genes (*blaCTX-M-9-like*, *blaCTX-M-15-like*, and *blaTEM*), Macrolide resistance genes (*ermB* and *ermC*), Quinolones resistance genes (*qnrD*, *qnrS* and *oqxB*), Sulfonamide resistance genes (*sul1*, *sul2* and *sul3*), the Aminoglycosides resistance gene *aadD* and Multidrug resistance genes (*acrA* and *acrB*). For the amplification process, 0.5 μL of purified DNA was used in a real-time PCR (qPCR) reaction containing 5 μL of iQ™ SYBR^®^ *Green* Supermix (Bio-Rad, Hercules, CA, USA) and 0.4 μL of each primer (10 μM), making up a final volume of 10 μL. For each gene analyzed, positive controls consisted of environmental samples previously validated by Sanger sequencing as containing the respective resistance genes [19]. A negative template control, composed of the PCR mixture and RNAse-free water in place of DNA, was included to check for potential contamination. The melting temperatures of ARGs were carefully analyzed, and amplification products outside the expected range were considered negative to minimize the risk of false positives. In addition, PCR products obtained in this study were confirmed by Sanger sequencing to ensure the reliability of the results by BLAST (https://blast.ncbi.nlm.nih.gov/Blast.cgi accessed 27th July 2025). The thermal cycling conditions were as follows: an initial denaturation step at 95 °C for 10 min; 40 cycles of denaturation at 95 °C for 10 s, annealing at a gene-specific temperature for 30 s, and extension at 72 °C for 30 s; followed by a final extension at 72 °C for 10 min and a hold at 4 °C. Primer sequences, annealing temperatures and expected product sizes for each gene are provided in Table 1. PCR products were separated on a 2% agarose gel prepared in 1× TAE buffer (0.04 mol/L Tris-acetate, 0.001 mol/L EDTA, pH 8.0), stained with GreenSafe (Nzytech, Lisbon, Portugal), and visualized under UV light.

### 2.4. Statistical Analysis

Each sample was examined for the presence or absence of ARGs, and the results were reported as the proportion of positive samples out of the total analyzed, along with the corresponding 95% confidence interval (95% CI). Data processing and analysis were carried out using Microsoft Excel^®^ for Microsoft 365 MSO (Redmond, WA, USA), and Fisher’s exact test was used to assess differences in the presence of antimicrobial resistance genes and classes between farms using RStudio version 4.4.2 (Boston, MA, USA).

## 3. Results

**Figure 1:**
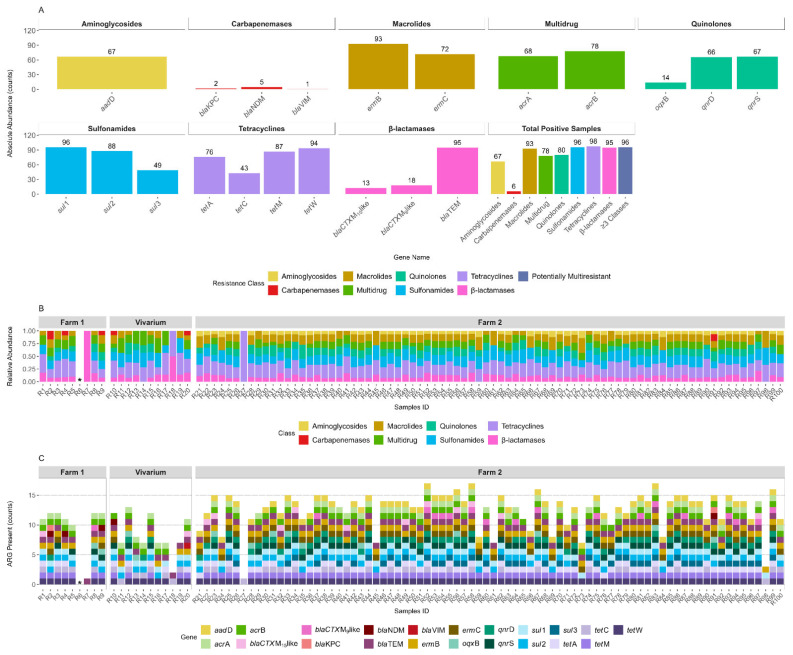
Abundance and distribution of antibiotic resistance genes (ARGs) across sampling sites and environments. (**A**) Absolute abundance of detected ARGs grouped by resistance class: aminoglycosides, carbapenemases, macrolides, multidrug resistance, quinolones, sulfonamides, tetracyclines and β-lactamases. For each gene within a class, bar height represents the total number of detections across all samples, with the numeric value indicating detection count. The rightmost panel summarizes the number of positive samples per resistance class and samples carrying ARGs from three or more classes. (**B**) Relative abundance of ARG classes in each sample across Farm 1, Vivarium and Farm 2 environments. Each bar represents the proportional composition of ARG classes within a single sample. Samples are sorted by source and sample ID. (**C**) ARG richness per sample, shown as the total count of distinct ARGs detected per sample. Colored segments within each bar represent specific ARGs, with colors corresponding to resistance classes as shown in the legend. * Represents the absence of ARGs.

A comprehensive analysis of rabbit stool samples by PCR revealed a widespread and diverse set of ARGs encompassing multiple antibiotic resistance classes (Figure 1A). The most abundant resistance genes were detected within the tetracycline, sulfonamide and β-lactamase classes. Among tetracycline resistance genes, *tetA*, *tetM* and *tetW* were highly prevalent, being present in 94, 87 and 76 samples, respectively. Sulfonamide resistance genes *sul1* (*n* = 96), *sul2* (*n* = 88), and *sul3* (*n* = 49) were also frequently detected.

Within the β-lactamase class, *blaTEM* was the most abundant gene (*n* = 95), followed by *blaCTX-M-15like* (*n* = 18) and *blaCTX-M-9like* (*n* = 13). Macrolide resistance was primarily driven by *ermB* (*n* = 93) and *ermC* (*n* = 72), while multidrug efflux pump genes *acrA* (*n* = 68) and *acrB* (*n* = 78), were detected at similar frequencies.

Aminoglycoside resistance was represented by the gene *aadD* (*n* = 67), whereas quinolone resistance genes *qnrB*, *oqxB*, and *qnrS* were identified at similar levels (*n* = 66–67). In contrast, carbapenemase genes were scarce, with *blaKPC* (*n* = 5), *blaNDM* (*n* = 2), and *blaVIM* (*n* = 1) detected at low frequencies, indicating limited but non-negligible dissemination of resistance to last-resort antibiotics.

The total number of positive samples per ARG class showed high levels of resistance gene diversity in nearly all samples, with the majority (93–98%) testing positive for genes in the tetracycline, sulfonamide and β-lactamase classes. Multidrug and macrolide resistance genes were also widespread, detected in 78% and 80% of samples, respectively. Only six percent of samples harbored carbapenemase genes, underscoring their relative rarity. Overall, 96% of the samples carried more ARG from three or more antibiotic classes, falling in the scope of multiresistant potential.

The relative abundance of ARGs across samples, normalized to total ARG content (Figure 1B), demonstrated consistent compositional patterns within and between sampling sources. Samples from both Farm 1 and Farm 2 exhibited relatively even distributions across resistance classes, although tetracycline, sulfonamide, and β-lactamase genes typically accounted for the largest proportional shares. In contrast, samples from vivarium animals displayed a slightly lower representation of multidrug and macrolide resistance genes.

Despite variations in individual gene presence, the class-level profiles remained relatively stable across management conditions, suggesting a core resistome structure shared across different environments.

Antibitotic resistance genes (ARG) richness, defined as the number of distinct ARGs detected per sample (Figure 1C), ranged from zero to 17 genes. Samples from Farm 2 exhibited the highest overall ARG diversity, with many samples presenting 12 or more distinct ARGs. In contrast, vivarium-derived samples displayed a lower richness (typically under 10 ARGs per sample). Farm 1 showed intermediate values but with considerable inter-sample variability, exception for sample R6 that did not harbor any ARG. In total, the number of ARG detections across all samples was *n* = 81 for Farm 1, *n* = 93 for the vivarium and *n* = 1018 for Farm 2.

Analysis of antimicrobial resistance across the three farms revealed significant variation at both the class and gene levels. Among resistance classes, aminoglycosides, carbapenemases, macrolides, quinolones, sulfonamides and tetracyclines showed adjusted *p* values < 0.05 indicating uneven distribution across farms while multidrug resistance genes and β-lactamases showed adjusted *p* values > 0.05 suggesting similar prevalence between farms. At the gene level several determinants including *aadD*, *blaNDM*, *ermB, ermC*, *qnrD*, *qnrS*, *sul1*, *sul3*, *tetA*, *tetC*, *tetM* and *tetW* had adjusted *p* values < 0.05 indicating significant differences between farms with *aadD*, *ermC*, *sul3*, *qnrD* and *ermB* exhibiting particularly strong associations. In contrast *acrA*, *acrB*, *blaCTX M 15 like*, *blaCTX M 9 like*, *blaKPC*, *blaTEM*, *blaVIM*, *oqxB* and *sul2* had adjusted *p* values > 0.05 consistent with a more stable distribution across the three farming environments.

## 4. Discussion

The present study characterizes ARGs in rabbit stool samples collected from both farming environments and laboratory facilities in Sumy region, Ukraine. The findings reveal a rich and diverse profile of ARGs, dominated by those conferring resistance to tetracyclines, sulfonamides, β-lactams, and macrolides. These results confirm a widespread presence of resistance determinants in rabbit stool, mirroring patterns observed in other livestock, and likely shaped by ongoing antimicrobial usage in animal production systems [21,22,23,24].

In commercial rabbit farming, three bacterial pathogens stand out due to their prevalence and clinical impact namely the most common, *Pasteurella multocida*, *Escherichia coli*, and *Staphylococcus aureus* [1,25,26]. These bacteria are responsible for respiratory, enteric and cutaneous infections, respectively [27,28,29], and are commonly treated with antibiotics from the same classes in which we detected a high prevalence of ARGs (tetracyclines, β-lactams, sulfonamides, and macrolides). Providing biological context to our findings, as the frequent therapeutic use of these antimicrobials against such pathogens in rabbits may have contributed to the selective pressure shaping the resistome profile observed in this study. Although information on antibiotic use in Ukraine cuniculture is scarce, previous studies reported the widespread use of tetracyclines, β-lactamase, sulfonamides, macrolides, aminoglycosides and quinolones [29,30].

The highest prevalence of ARGs corresponds to the tetracycline class, detected in almost every sample. These findings suggest selective pressure and align with the literature, which identifies tetracyclines as among the most frequently used antibiotics against diverse pathogens in this animal species. This prevalence likely stems from the extensive use of these antibiotic classes in Ukrainian agriculture. Tetracyclines represent one of the most extensively used classes of antimicrobials in veterinary medicine worldwide and hold a particularly dominant position in Ukrainian agriculture, where they account for the largest proportion of veterinary antimicrobial sales [31]. Although antimicrobial use has been regulated since 2006, some authors suggest that reductions have been modest [18,32,33]. Furthermore, recent conflict in Ukraine may have facilitated increased antibiotic acquisition and use, potentially weakening regulatory oversight. In some cases, therapeutic use has been replaced by metaphylactic or prophylaxis administration.

The high detection rate of *blaTEM*, a gene encoding resistance to commonly used β-lactam antibiotics and the presence of extended-spectrum β-lactamase (ESBL) genes like *blaCTX-M-15like* and *blaCTX-M-9like*, raise particular concern [34,35]. Although these ESBLs were found with less prevalence (*n* = 13 and *n* = 18, respectively), their detection in a non-priority species such as rabbits highlights a potentially overlooked reservoir for resistance genes of high clinical importance. The co-occurrence of macrolide resistance genes (*ermB*, *ermC*) and multidrug efflux pumps (*acrA*, *acrB*) in a substantial proportion of samples further indicates that multidrug resistance profiles may be maintained within this animal population. Indeed, 96% of samples harbored ARGs from three or more resistance classes, suggesting that many of the microbial communities associated with these animals may already possess broad resistance phenotypes.

Although carbapenemase genes were detected at low frequencies, their presence should not be overlooked. The identification of *blaKPC*, *blaNDM*, and *blaVIM*, albeit in a handful of samples, is troubling, given that carbapenems are not licensed for veterinary medicine due to risks to public health [36]. Their presence may be attributed to co-selection via mobile genetic elements or environmental contamination linked to human or industrial sources [37,38]. Given the mobility of these genes and their association with hospital-acquired infections, even low-level detection in animal reservoirs demands attention [39].

The relative abundance data confirmed that tetracycline, sulfonamide, and β-lactam ARGs consistently dominated the resistome across all sources, including both farms and laboratory animals. This distribution likely reflects differences in the frequency of use of these antibiotic classes in rabbit husbandry, the long-term persistence of their resistance determinants in bacterial populations, and the frequent association of tetracycline and sulfonamide resistance genes with mobile genetic elements that facilitate their dissemination [40]. Laboratory animals showed a slightly lower representation of macrolide and multidrug resistance genes, likely reflecting minimal or no direct antibiotic exposure in controlled settings. While class-level resistome profiles were broadly consistent between farms, supporting the existence of a core resistome maintained across environments, some variation emerged. For instance, aminoglycoside resistance genes were detected only in Farm 2, possibly indicating differences in antibiotic administration. However, due to the lack of antibiotic usage data, this remains speculative.

The number of ARGs per sample ranged from 0 to 17. Farm 2 exhibited the highest richness, likely owing to greater environmental microbial diversity, the number of samples being superior or more frequent antimicrobial exposure. Animals at this farm cohabit with other livestock, share manure, and receive the same feed, which may contribute to prominent diversity. In contrast, Farm 1 showed more variable ARG profiles, while laboratory animals presented the lowest diversity, consistent with their limited environmental exposure. These findings hold several important implications for public health, particularly considering the role of rabbits in the Ukrainian food system [9]. In 2023 rabbit meat production reached an estimated 8.400 tones according to FAOSTAT [41]. Because rabbit meat is often processed outside industrial slaughter systems usually in private households, increasing the risk of fecal contamination and subsequent human exposure to resistant bacteria or resistance genes [42]. Inadequate hygiene during slaughter or insufficient cooking could lead to the ingestion of ARG-carrying bacteria, with potential colonization of the human gut microbiome [43]. Farm workers and individuals in close contact with rabbits are particularly at risk of direct transmission through handling of animals, bedding, or feed [44].

In addition to direct contact with rabbits or their environment, humans may be exposed to these ARGs through multiple indirect pathways. Consumption of meat or other products, especially if processed under suboptimal hygiene conditions or insufficiently cooked, can facilitate the ingestion of ARG-carrying bacteria [45]. Environmental dissemination is also plausible, as manure used as fertilizer or runoff from farms may contaminate soil and water sources, creating opportunities for ARGs to enter the broader human microbiome [46,47]. Occupational exposure remains a critical route, particularly for farm workers and laboratory personnel handling animals, bedding, or feed, where repeated contact could increase the likelihood of colonization or transfer of resistant bacteria [44,48,49].Comparing our findings to existing studies reveals a consistent pattern of dominant resistance classes across different regions. In Italy [50], the most prevalent antibiotic resistance classes in *S. aureus* isolates from rabbits were tetracyclines (95.8%), macrolides (93.8%), and quinolones (63.5%). These data closely mirror our own results, where tetracycline and macrolide resistance genes such as tetM, tetW, ermB, and ermC were among the most frequently detected, suggesting the presence of a conserved resistome structure within rabbit production systems.

Similarly, studies in Portugal have reported widespread *E. coli* resistance to multiple key antibiotics, including tetracycline (91.6%), ampicillin (100%), aztreonam (97.8%), streptomycin (93.7%), tobramycin (64.58%), trimethoprim/sulfamethoxazole (75%), amoxicillin–clavulanic acid (54.16%), and chloramphenicol (72.9%) [24]. These phenotypic resistance patterns correspond closely with our detection of sulfonamide genes (*sul1*, *sul2*, *sul3*), β-lactamase genes (*blaTEM, blaCTX-M-15like*, *blaCTX-M-9like*), and aminoglycoside resistance genes such as *aadD*. The identification of extended-spectrum β-lactamase (ESBL) genes like *blaCTX-M* variants in both our study and the Portuguese literature further underscores the presence and circulation of clinically significant resistance determinants in rabbit-associated microbial communities.

The identification of multiple clinically significant ARGs, including ESBLs and even carbapenemases, supports the potential for these resistance determinants to spread to human pathogens through direct contact with rabbits or their feces during husbandry and handling, or via the food chain through the consumption of contaminated meat products [51]. The possibility of horizontal gene transfer, particularly through plasmids or integrative elements not characterized in this study, remains high and warrants further investigation. While phenotypic resistance, gene expression, and genomic context were beyond the scope of this study, the co-detection of multiple ARGs within individual samples suggests the involvement of mobile genetic elements that facilitate gene clustering and horizontal transfer. This underscores the need to address these gaps in future research.

Despite the interesting findings of this study and the notable prevalence of ARGs, several limitations must be addressed in future research. Namely, the aim of this study was limited to the assessment of presence or absence of ARGs but not quantification of their abundance. While this approach is suitable for assessing the diversity and distribution of resistance genes across farms, it does not capture differences in gene load or relative prevalence, which may have important implications for antimicrobial resistance risk. Future work should therefore incorporate abundance-based analyses to complement presence/absence data and provide a more quantitative understanding of the resistome. The stronger focus on Farm 2 reflects the greater availability of animals and practical constraints at the time of sampling; however, this imbalance may limit the representativeness of the findings. To estimate ARG distribution patterns across the Ukrainian cuniculture industry, extensive geographical sampling is needed, increasing both the number of samples and sampling locations. Finally, to gain a more comprehensive understanding of the gut resistome, metagenomic approaches using next-generation sequencing should be employed, as they could provide a broader and more detailed characterization of ARGs.

In the context of Ukraine’s evolving veterinary regulatory framework, this study provides molecular evidence that rabbits carry a diverse array of antimicrobial resistance genes (ARGs), including several with known clinical relevance. Although the study is limited by the absence of phenotypic resistance profiles and records of antimicrobial usage, the breadth of ARGs detected highlights the need to include non-traditional livestock species such as rabbits in national AMR surveillance systems. Current monitoring efforts primarily target major food-producing animals, while rabbits are often raised in small-scale or informal production systems that fall outside the scope of regulatory oversight. This gap presents a risk, as these animals may contribute to the dissemination of resistance. Expanding surveillance to include rabbit farming would enable earlier identification of high-risk resistance patterns and support more effective mitigation strategies. It is also essential to improve traceability of antimicrobial use in rabbit production and to encourage practices that align with international standards for responsible antimicrobial stewardship. These steps are necessary to better assess AMR risks from small livestock and reduce the potential for transmission to human and environmental systems.

## 5. Conclusions

This study reveals a high prevalence and diversity of ARGs in rabbit farms in northeastern Ukraine, highlighting notable risks to both animal and public health. Nearly every fecal sample harbored ARGs from multiple antibiotic classes, including clinically significant genes such as *blaCTX-M*, *ermB*, and efflux pumps like *acrA* and *acrB*. Although genes conferring resistance to carbapenems were seldom detected, their presence is especially concerning, given their restricted use in veterinary medicine and their use as a last resort for a variety of different human bacterial infections. The consistency of ARG profiles across farms suggests a stable resistome shaped by ongoing antimicrobial pressure. Current veterinary oversight may be insufficient, particularly in small-scale or informal production systems.

These findings underscore the need for stronger antibiotic stewardship, targeted surveillance in non-traditional livestock, and One Health-aligned strategies to curb the spread of resistance and safeguard both human and animal health.

## Figures and Tables

**Table 1:** Sequences of primers, annealing temperatures, and expected sizes of ARGs associated with various classes of antibiotics.

Class	Gene	Sequence (5′ > 3′)	Annealing Temperature (°C)	Product Size (bp)	Reference
**Tetracyclines**	*tetA*	F:GCTACATCCTGCTTGCCTTC R:CATAGATCGCCGTGAAGAGG	60	210	[6]
	*tetC*	F:TGCAACTCGTAGGACAGGTG R:ACCAGTGACGAAGGCTTGAG	60	139	
	*tet* *M*	F:ACAGAAAGCTTATTATATAAC R:GGCGTGTCTATGATGTTCAC	51	171	
	*tet* *W*	F:GAGAGCCTGCTATATGCCAGC R:GGGCGTATCCACAATGTTAAC	60	168	
Carbapenemases	*bla* *NDM*	F:CAGTCGCTTCCAACGGTTTG R: AACGCATTGGCATAAGTCGC	60	217	[20]
	*bla* *VIM*	F:AAAACACAGCGGCACTTCTC R: AATCTCGTTCCCCTCTACCTC	60	182	
	*bla* *KPC*	F:CATTCGCTAAACTCGAACAGG R: TTTTGCCGTAACGGATGG	60	201	
Extended-spectrum β-lactamases (ESBL)	*blaCTX-M-15like*	F:GCTGGTGACATGGATGAAAG R: TAGGTTGAGGCTGGGTGAAG	60	87	[20]
	*blaCTX-M-9like*	F:GTTGGTGACGTGGCTCAAAG R: GTTGCGGCTGGGTAAAATAG	60	89	
	*bla* *TEM*	F:TCTGACAACGATCGGAGGAC R: TGCCGGGAAGCTAGAGTAAG	60	86	
Macrolides	*erm* *B*	F:AGGGTTGCTCTTGCACACTC R: CTGTGGTATGGCGGGTAAGT	58	119	[6]
	*erm* *C*	F:GAAATCGGCTCAGGAAAAGG R: TAGCAAACCCGTATTCCACG	56	292	
Quinolones	*qnr* *D*	F:GCTGGTGACATGGATGAAAG R: TAGGTTGAGGCTGGGTGAAG	57	373	
	*qnr* *S*	F:GTTGGTGACGTGGCTCAAAG R: GTTGCGGCTGGGTAAAATAG	55.3	134	
	*oqx* *B*	F:TCTGACAACGATCGGAGGAC R: TGCCGGGAAGCTAGAGTAAG	60	131	
Sulfonamides	*sul* *1*	F:TGTCGAACCTTCAAAAGCTG R: TGGACCCAGATCCTTTACAG	60	113	
	*sul* *2*	F:ATCTGCCAAACTCGTCGTTA R: CAATGTGATCCATGATGTCG	60	89	
	*sul* *3*	F:AGGCTTGGCAAAGTCAGATT R: CACCAGCCTCAACTAAAGCA	57	152	
Aminoglycosides	*aad* *D*	F:ATGGGGATGATGTTAAGGCT R: TCACTTCCACCTTCCACTCA	55	153	
Multi-drug	*acr* *A*	F:CTCTCAGGCAGCTTAGCCCTAA R: TGCAGAGGTTCAGTTTTGACTGTT	60	107	
	*acr* *B*	F:GGTCGATTCCGTTCTCCGTTA R:CTACCTGGAAGTAAACGTCATTGGT	60	105	

## Data Availability

The original contributions presented in this study are included in the article. Further inquiries can be directed at the corresponding author.

## References

[1] Flynn C.E., Guarner J. (2023). Emerging Antimicrobial Resistance. Mod. Pathol..

[2] Rizzo A., Piccinno M., Lillo E., Carbonari A., Jirillo F., Sciorsci R.L. (2023). Antimicrobial Resistance and Current Alternatives in Veterinary Practice: A Review. Curr. Pharm. Des..

[3] Zalewska M., Błażejewska A., Czapko A., Popowska M. (2021). Antibiotics and Antibiotic Resistance Genes in Animal Manure—Consequences of Its Application in Agriculture. Front. Microbiol..

[4] Scott L.C., Menzies P.I. (2011). Antimicrobial Resistance and Small Ruminant Veterinary Practice. Vet. Clin. N. Am. Food Anim. Pract..

[5] Mulchandani R., Wang Y., Gilbert M., Van Boeckel T.P. (2023). Global Trends in Antimicrobial Use in Food-Producing Animals: 2020 to 2030. PLOS Glob. Public Health.

[6] Chen B., Hao L., Guo X., Wang N., Ye B. (2015). Prevalence of Antibiotic Resistance Genes of Wastewater and Surface Water in Livestock Farms of Jiangsu Province, China. Environ. Sci. Pollut. Res. Int..

[7] Luiken R.E., Heederik D.J., Scherpenisse P., Van Gompel L., van Heijnsbergen E., Greve G.D., Jongerius-Gortemaker B.G., Tersteeg-Zijderveld M.H., Fischer J., Juraschek K. (2022). Determinants for Antimicrobial Resistance Genes in Farm Dust on 333 Poultry and Pig Farms in Nine European Countries. Environ. Res..

[8] Cao H., Bougouffa S., Park T.-J., Lau A., Tong M.-K., Chow K.-H., Ho P.-L. (2022). Sharing of Antimicrobial Resistance Genes between Humans and Food Animals. mSystems.

[9] Zamaratskaia G., Havrysh O., Korzeniowska M., Getya A. (2023). Potential and Limitations of Rabbit Meat in Maintaining Food Security in Ukraine. Meat Sci..

[10] Szendrő K., Szabó-Szentgróti E., Szigeti O. (2020). Consumers’ Attitude to Consumption of Rabbit Meat in Eight Countries Depending on the Production Method and Its Purchase Form. Foods.

[11] Kotelevych V.A. (2019). Veterinary and Sanitary Evaluation of Rabbit as an Important Reserve of Dietary Products. Sci. Messenger LNU Vet. Med. Biotechnol. Ser. Vet. Sci..

[12] Falcão-e-Cunha L., Solla L.C., Maertens L., Marounek M., Pinheiro V., Freire J., Mourão J.L. (2007). Alternatives to Antibiotic Growth Promoters in Rabbit Feeding: A Review. World Rabbit. Sci..

[13] Agnoletti F., Brunetta R., Bano L., Drigo I., Mazzolini E. (2018). Longitudinal Study on Antimicrobial Consumption and Resistance in Rabbit Farming. Int. J. Antimicrob. Agents.

[14] Crovato S., Menegon F., Mascarello G., Pinto A., Nadin A., Piovan G., Ricaldi G., Di Martino G., Pozza G. (2023). Development of a Training Strategy Aimed at Increasing Veterinarians’ Awareness of the Proper Use of Antibiotics on Rabbit Farms. Animals.

[15] Nielsen S.S., Bicout D.J., Calistri P., Canali E., Drewe J.A., Garin-Bastuji B., Gonzales Rojas J.L., Gortazar Schmidt C., Herskin M., EFSA Panel on Animal Health and Welfare (AHAW) (2021). Assessment of Animal Diseases Caused by Bacteria Resistant to Antimicrobials: Rabbits. EFSA J..

[16] Drigo I., Mazzolini E., Bacchin C., Tonon E., Puiatti C., Bano L., Spigaglia P., Barbanti F., Agnoletti F. (2015). Molecular Characterization and Antimicrobial Susceptibility of *Clostridium difficile* Isolated from Rabbits Raised for Meat Production. Vet. Microbiol..

[17] Agnoletti F., Mazzolini E., Bacchin C., Bano L., Berto G., Rigoli R., Muffato G., Coato P., Tonon E., Drigo I. (2014). First Reporting of Methicillin-Resistant *Staphylococcus aureus* (MRSA) ST398 in an Industrial Rabbit Holding and in Farm-Related People. Vet. Microbiol..

[18] Jeżak K., Kozajda A. (2022). Occurrence and Spread of Antibiotic-Resistant Bacteria on Animal Farms and in Their Vicinity in Poland and Ukraine—Review. Environ. Sci. Pollut. Res..

[19] Bento J.T., Gomes-Gonçalves S., Cruz R., Esteves F., Baptista A.L., Aires Pereira M., Caseiro P., Carreira P., Figueira L., Mesquita J.R. (2025). The Prevalence of Antimicrobial Resistance Genes in the Environments of Small Ruminant Farms from Central Portugal. Antibiotics.

[20] Shi X., Li Y., Yang Y., Shen Z., Cai C., Wang Y., Walsh T.R., Shen J., Wu Y., Wang S. (2021). High Prevalence and Persistence of Carbapenem and Colistin Resistance in Livestock Farm Environments in China. J. Hazard. Mater..

[21] Poulin-Laprade D., Brouard J.-S., Gagnon N., Turcotte A., Langlois A., Matte J.J., Carrillo C.D., Zaheer R., McAllister T.A., Topp E. (2021). Resistance Determinants and Their Genetic Context in Enterobacteria from a Longitudinal Study of Pigs Reared under Various Husbandry Conditions. Appl. Environ. Microbiol..

[22] Guo K., Zhao Y., Cui L., Cao Z., Zhang F., Wang X., Feng J., Dai M. (2021). The Influencing Factors of Bacterial Resistance Related to Livestock Farm: Sources and Mechanisms. Front. Anim. Sci..

[23] He Y., Yuan Q., Mathieu J., Stadler L., Senehi N., Sun R., Alvarez P.J.J. (2020). Antibiotic Resistance Genes from Livestock Waste: Occurrence, Dissemination, and Treatment. npj Clean Water.

[24] Silva A., Silva V., Tavares T., López M., Rojo-Bezares B., Pereira J.E., Falco V., Valentão P., Igrejas G., Sáenz Y. (2024). Rabbits as a Reservoir of Multidrug-Resistant Escherichia Coli: Clonal Lineages and Public Health Impact. Antibiotics.

[25] El-Ghany A., Wafaa A. (2023). Pasteurellosis: A Significant Bacterial Disease in Rabbit Production. Vet. Stanica.

[26] Solans L., Arnal J.L., Sanz C., Benito A., Chacón G., Alzuguren O., Fernández A.B. (2019). Rabbit Enteropathies on Commercial Farms in the Iberian Peninsula: Etiological Agents Identified in 2018–2019. Animals.

[27] Deeb B.J. (2004). Respiratory Disease and Pasteurellosis. Ferrets, Rabbits, and Rodents.

[28] Prescott J.F. (1978). *Escherichia coli* and Diarrhoea in the Rabbit. Vet. Pathol..

[29] Vancraeynest D., Hermans K., Martel A., Vaneechoutte M., Devriese L.A., Haesebrouck F. (2004). Antimicrobial Resistance and Resistance Genes in *Staphylococcus aureus* Strains from Rabbits. Vet. Microbiol..

[30] Sun C., Wang Z., Li Y., Huang J. (2024). Antibiotic Resistance Spectrums of *Escherichia coli* and *Enterococcus* spp. Strains against Commonly Used Antimicrobials from Commercial Meat-Rabbit Farms in Chengdu City, Southwest China. Front. Vet. Sci..

[31] Kosenko Y.M., Ostapiv N.V., Zaruma L.E. (2024). Safety of Tetracyclines for Public Health and the Environment. Sci. Tech. Bull. State Sci. Res. Control Inst. Vet. Med. Prod. Fodd. Addit. Inst. Anim. Biol..

[32] Argudín M.A., Deplano A., Meghraoui A., Dodémont M., Heinrichs A., Denis O., Nonhoff C., Roisin S. (2017). Bacteria from Animals as a Pool of Antimicrobial Resistance Genes. Antibiotics.

[33] Woolhouse M., Ward M., van Bunnik B., Farrar J. (2015). Antimicrobial Resistance in Humans, Livestock and the Wider Environment. Philos. Trans. R. Soc. B Biol. Sci..

[34] Mroczkowska J.E., Barlow M. (2008). Fitness Trade-Offs in bla_TEM_ Evolution. Antimicrob. Agents Chemother..

[35] Husna A., Rahman M.M., Badruzzaman A.T.M., Sikder M.H., Islam M.R., Rahman M.T., Alam J., Ashour H.M. (2023). Extended-Spectrum β-Lactamases (ESBL): Challenges and Opportunities. Biomedicines.

[36] Ramírez-Castillo F.Y., Guerrero-Barrera A.L., Avelar-González F.J. (2023). An Overview of Carbapenem-Resistant Organisms from Food-Producing Animals, Seafood, Aquaculture, Companion Animals, and Wildlife. Front. Vet. Sci..

[37] Ramsamy Y., Mlisana K.P., Amoako D.G., Abia A.L.K., Ismail A., Allam M., Mbanga J., Singh R., Essack S.Y. (2022). Mobile Genetic Elements-Mediated Enterobacterales-Associated Carbapenemase Antibiotic Resistance Genes Propagation between the Environment and Humans: A One Health South African Study. Sci. Total Environ..

[38] Mills M.C., Lee J. (2019). The Threat of Carbapenem-Resistant Bacteria in the Environment: Evidence of Widespread Contamination of Reservoirs at a Global Scale. Environ. Pollut..

[39] Aldali H.J., Khan A., Alshehri A.A., Aldali J.A., Meo S.A., Hindi A., Elsokkary E.M. (2023). Hospital-Acquired Infections Caused by Carbapenem-Resistant Enterobacteriaceae: An Observational Study. Microorganisms.

[40] Mu Q., Li J., Sun Y., Mao D., Wang Q., Luo Y. (2015). Occurrence of Sulfonamide-, Tetracycline-, Plasmid-Mediated Quinolone- and Macrolide-Resistance Genes in Livestock Feedlots in Northern China. Environ. Sci. Pollut. Res..

[41] FAOSTAT. https://www.fao.org/faostat/en/#data/QCL/visualize.

[42] Zhou Z., Chen H. (2024). Evaluating Human Exposure to Antibiotic Resistance Genes. Biosaf. Health.

[43] Farrukh M., Munawar A., Nawaz Z., Hussain N., Hafeez A.B., Szweda P. (2025). Antibiotic Resistance and Preventive Strategies in Foodborne Pathogenic Bacteria: A Comprehensive Review. Food Sci. Biotechnol..

[44] Castillo Neyra R., Vegosen L., Davis M.F., Price L., Silbergeld E.K. (2012). Antimicrobial-Resistant Bacteria: An Unrecognized Work-Related Risk in Food Animal Production. Saf. Health Work..

[45] Conceição S., Queiroga M.C., Laranjo M. (2023). Antimicrobial Resistance in Bacteria from Meat and Meat Products: A One Health Perspective. Microorganisms.

[46] Tiedje J.M., Fu Y., Mei Z., Schäffer A., Dou Q., Amelung W., Elsner M., Adu-Gyamfi J., Heng L., Virta M. (2023). Antibiotic Resistance Genes in Food Production Systems Support One Health Opinions. Curr. Opin. Environ. Sci. Health.

[47] Seyoum M.M., Ashworth A.J., Feye K.M., Ricke S.C., Owens P.R., Moore P.A., Savin M. (2023). Long-Term Impacts of Conservation Pasture Management in Manuresheds on System-Level Microbiome and Antibiotic Resistance Genes. Front. Microbiol..

[48] Karwowska E. (2024). Antibiotic Resistance in the Farming Environment. Appl. Sci..

[49] Samreen, Ahmad I., Malak H.A., Abulreesh H.H. (2021). Environmental Antimicrobial Resistance and Its Drivers: A Potential Threat to Public Health. J. Glob. Antimicrob. Resist..

[50] Attili A.-R., Bellato A., Robino P., Galosi L., Papeschi C., Rossi G., Fileni E., Linardi M., Cuteri V., Chiesa F. (2020). Analysis of the Antibiotic Resistance Profiles in Methicillin-Sensitive S. Aureus Pathotypes Isolated on a Commercial Rabbit Farm in Italy. Antibiotics.

[51] Sagar P., Aseem A., Banjara S.K., Veleri S. (2023). The Role of Food Chain in Antimicrobial Resistance Spread and One Health Approach to Reduce Risks. Int. J. Food Microbiol..

